# Joint hypermobility is not positively associated with prevalent multiple joint osteoarthritis: a cross-sectional study of older adults

**DOI:** 10.1186/s12891-019-2550-z

**Published:** 2019-04-11

**Authors:** Terese R. Gullo, Yvonne M. Golightly, Portia Flowers, Joanne M. Jordan, Jordan B. Renner, Todd A. Schwartz, Virginia B. Kraus, Marian T. Hannan, Rebecca J. Cleveland, Amanda E. Nelson

**Affiliations:** 1 The Ohio State University College of Medicine, Columbus, OH USA; 2 Thurston Arthritis Research Center, University of North Carolina, 3300 Doc J. Thurston Building, Campus Box #7280, Chapel Hill, NC 27599-7280 USA; 3 Department of Epidemiology, Gillings School of Global Public Health, University of North Carolina, Chapel Hill, NC USA; 4 Injury Prevention Research Center, University of North Carolina, Chapel Hill, USA; 5 Division of Physical Therapy, University of North Carolina, Chapel Hill, NC USA; 6 Department of Medicine, University of North Carolina, Chapel Hill, USA; 7 Department of Radiology, University of North Carolina, Chapel Hill, NC USA; 8 Duke University School of Medicine, Durham, NC USA; 9 Institute for Aging Research, Hebrew SeniorLife, and Harvard Medical School, Boston, MA USA; 10 Department of Biostatistics, Gillings School of Global Public Health, University of North Carolina, Chapel Hill, NC USA

**Keywords:** Hypermobility, Generalized osteoarthritis, Polyarticular osteoarthritis

## Abstract

**Background:**

This cross-sectional study evaluated associations of joint hypermobility and multiple joint osteoarthritis (MJOA) in a community-based cohort of adults 45+ years of age.

**Methods:**

MJOA and joint hypermobility data were from 1677 participants (mean age 69 years, 68% women) who completed research clinic visits during 2003–2010. Prevalent MJOA was defined in four ways. Radiographic OA (rOA) was defined as Kellgren-Lawrence (KL) > 2 at any included study joint; symptomatic OA (sxOA) required both symptoms and rOA in a joint. Joint hypermobility was defined as a Beighton score of > 4. Separate logistic regression models were used to estimate odds ratios (OR) between joint hypermobility and each MJOA definition, adjusting for age, sex, race, body mass index, and baseline visit.

**Results:**

In this cohort, 4% had Beighton score > 4 and 63% met any definition of MJOA. Joint hypermobility was associated with significantly lower odds of radiographic and symptomatic MJOA-1 (multiple joint OA-definition 1: involvement of > 1 IP (interphalangeal) nodes and > 2 sites of hip, knee, and spine; 74 and 58% lower, respectively). However, for the other MJOA definitions (i.e., MJOA-2:involvement of > 2 IP joints, > 1 carpometacarpal [CMC] joints, and knee or hip sites; MJOA-3: involvement of > 5 joint sites from among distal interphalangeal, proximal interphalangeal, CMC, hip, knee, or spine sites; and MJOA-4:involvement of > 2 lower body sites (hip, knee, or spine), there were no statistically significant associations. For associations between site-specific hypermobility and any MJOA definition, most adjusted ORs were less than one, but few were statistically significant.

**Conclusions:**

Overall, joint hypermobility was not positively associated with any definition of prevalent MJOA in this cohort, and an inverse association existed with one definition of MJOA. Longitudinal studies are needed to determine the contribution of hypermobility to the incidence and progression of MJOA outcomes.

## Background

Osteoarthritis (OA) is a common and debilitating disease with a large public health burden in the United States and globally [1]. OA development and progression can be influenced by biomechanical factors (e.g., joint injury, obesity) that change joint structures, alignment, motion, and loading, Joint hypermobility, broadly defined as range of motion of the joint that is greater than normal, may also influence OA development and progression [2]. The laxity of ligaments can contribute to increased range of motion, potentially leading to a hypermobile joint. Abnormalities in collagen and elastin may contribute to ligamentous laxity, with less severe defects occurring in isolated forms of joint hypermobility syndrome compared to heritable collagen diseases, such as Ehlers-Danlos syndrome [3]. Hypermobility may contribute to joint injury [4] (microtraumas over time from stresses to joint structures at extremes of range of motion or an increased susceptibility to a single major traumatic event), pain [5], and damage to atypical contact areas of cartilage tissue [6]. Due to this propensity for altered biomechanics and injury, joint hypermobility may be a unique risk factor for OA [7, 8].

Joint hypermobility, which occurs in 10–25% of adults depending on the population and hypermobility definition [9–12], is associated with female sex and young age, with the degree of joint range of motion decreasing as an individual ages [13]. Due to conflicting evidence in the literature to date, the relationship between hypermobility and OA is unclear. Multiple studies have found joint hypermobility to be related to arthralgia and OA of joints in the upper and lower body [2, 13–15], although these associations are not always consistent [16, 17]. More recent work has found a wide range of relationships between hypermobility and various joint sites affected by OA [18–21].

In the individual with OA, more than one joint site may be affected. Thus, it is appropriate to evaluate the association of generalized hypermobility (as measured by the Beighton score) not solely with single joint OA, but with the occurrence of multiple joint OA (MJOA) as well. Currently, the possible role of joint hypermobility in MJOA is not known. It is evident, however, that MJOA may represent a distinct etiology from mono-articular OA [22–27] and is associated with increased disease burden in the individual patient [28–35]. Accordingly, it should be considered separately when assessing for associated conditions, including joint hypermobility. Of note, MJOA is relatively understudied and lacks a precise definition in the literature. To account for this, a systematic review was conducted in conjunction with this study to arrive at a list of representative definitions to classify the condition and aid in data analysis [36]. In this study, we aimed to examine the association of literature-based MJOA definitions and joint hypermobility in a large community-based sample of adults: the Johnston County Osteoarthritis (JoCo OA) Project.

## Methods

### Summary of systematic review and MJOA definitions

From a list of 10 possible MJOA definitions derived from a systematic literature review [36], 4 were selected for this analysis based on relevance and available data (MJOA-1 through − 4). MJOA-1 is involvement of 1 or more interphalangeal (IP) nodes and 2 or more sites (where 1 site indicates 1 joint, i.e. right knee, left hip, etc.) including hip, knee, and spine; MJOA-2 is involvement of 2 or more IP joints, 1 or more carpometacarpal (CMC) joints, and knee or hip sites; MJOA-3 is involvement of 5 or more joint sites from among distal interphalangeal (DIP), proximal interphalangeal (PIP), CMC, hip, knee, or spine sites; and MJOA-4 is involvement of 2 or more lower body sites (hip, knee, or spine). In this analysis, all 4 definitions were considered based on radiographic and symptomatic criteria and, where applicable, presence of IP nodes. Data was analyzed separately based on participants: 1) meeting criteria for radiographic MJOA-1 through − 4 and 2) meeting criteria for symptomatic MJOA-1 through-4 (described in detail below).

### Study participants

Participants in this cross-sectional analysis completed a research clinic visit during 2003–2010 (the time period in which hypermobility measures were collected) as part of the JoCo OA Project, a community-based prospective cohort study of OA in African American and white men and women aged 45 years and older in Johnston County, North Carolina that has been previously described in detail [37]. The JoCo OA Project cohort has a high prevalence of sociodemographic groups at risk for poor health outcomes. This study was approved by the Institutional Review Boards of the University of North Carolina at Chapel Hill and the Centers for Disease Control and Prevention. All participants gave written informed consent at the time of recruitment and each research clinic visit assessment.

All participants underwent interviews, radiographic evaluation, and a standardized musculoskeletal examination including the hands, knees, and hips, by trained staff. Age, sex, and race were self-reported. Body mass index (BMI) was determined from height in cm and weight in kg measured at the research clinic visit. In addition to the Beighton exam detailed below, the examination included determination of bony enlargement (i.e. IP nodes) at each of the 30 hand joints, assessed as present or absent. Two staff members independently performed the hand exam in a subset of 40 randomly selected participants with agreement ranging from 0.57 to 0.97 for nodes and 0.86 to 0.97 for tenderness [36].

#### Radiographic OA

Participants were objectively evaluated for radiographic OA (rOA), using radiographs of the hands (posteroanterior), knees (weight-bearing fixed-flexion posteroanterior with a Synaflexer device), hips (supine anteroposterior) and lumbar spine (lateral) obtained in a standardized fashion and graded by a single, highly reliable (weighted intra-rater kappa was 0.9 for each of: knee and hip [38], hand [39], and spine [40]) bone and joint radiologist using the Kellgren-Lawrence grading (KL) scale. Knee, hip and hand joint rOA was defined for analysis as KL of at least 2. Hand rOA (by KL grade) was assessed at each individual hand joint (DIP, PIP, CMC, and metacarpophalangeal). Lumbar spine rOA was defined as disc space narrowing of grade 1 or above and presence of osteophytes of grade 2 or above in one or more lumbar spine levels.

#### Symptomatic OA

Participants with symptomatic OA had both multiple joint rOA according to the criteria above and symptoms in at least one joint site in each definition. For example, symptomatic MJOA-1 is defined as rOA of 1 or more IP nodes and 2 or more sites including the hip, knee, and spine in addition to symptoms in one or more of any of these sites. For the hands, symptoms were ascertained as tenderness on exam in each specific joint site, while for other sites it was based on a question in the form “on most days, do you have pain, aching, or stiffness in your [right/left knee, right/left hip, lower back]?”

#### Hypermobility

Data were collected for joint hypermobility using the Beighton Criteria. Considered a standard assessment in clinical settings, the Beighton Criteria has been used in multiple studies to evaluate the presence of joint hypermobility [2]. This assessment evaluates joint range of motion at 5 separate body sites by assessing a participant’s ability to perform the following 9 maneuvers: right and left passive dorsiflexion of the 5th finger 90° or more, right and left passive apposition of the thumb to the flexor aspect of the forearm, right and left elbow hyperextension 10° or more, right and left knee hyperextension 10° or more, and forward flexion of the trunk, with knees extended and both palms flat on the floor (for photographic depiction of these actions, see [2]). One point is allotted for completion of each maneuver, with the sum of all 9 items representing the total score (maximum score of 9, minimum 0) [13]. Among 40 randomly selected participants, inter-rater reliability of two trained examiners for each maneuver was high (κ > 0.80) [21]. Based on the literature, a Beighton score of 4 or above was considered general joint hypermobility [2]. Outcomes were assessed based on Beighton cutoffs of > 3 and > 4. Additionally, data were collected for individuals with hypermobility in individual joints as defined by each joint-specific maneuver above. This allowed consideration of measures for both generalized and localized joint hypermobility.

### Statistical analysis

The Beighton score assessed at the 2003–2010 research clinic visit was used to define joint hypermobility and was compared to OA data that were collected at that same time point. For analyses of MJOA and hypermobility, inclusion criteria included having non-missing values for Beighton and MJOA measures as well as covariates (age, sex, race, BMI and baseline visit). Descriptive population characteristics are expressed as percentages for categorical variables and means with standard deviation for continuous variables. Adjusted odds ratios (aOR) with 95% confidence intervals (CI) were calculated via separate logistic regression models for each MJOA definition, adjusted for the above covariates. As we were more interested in exploring potential associations in this work, we elected not to statistically adjust for multiple comparisons, but rather to account for this when interpreting the results.

## Results

**Fig. 1:**
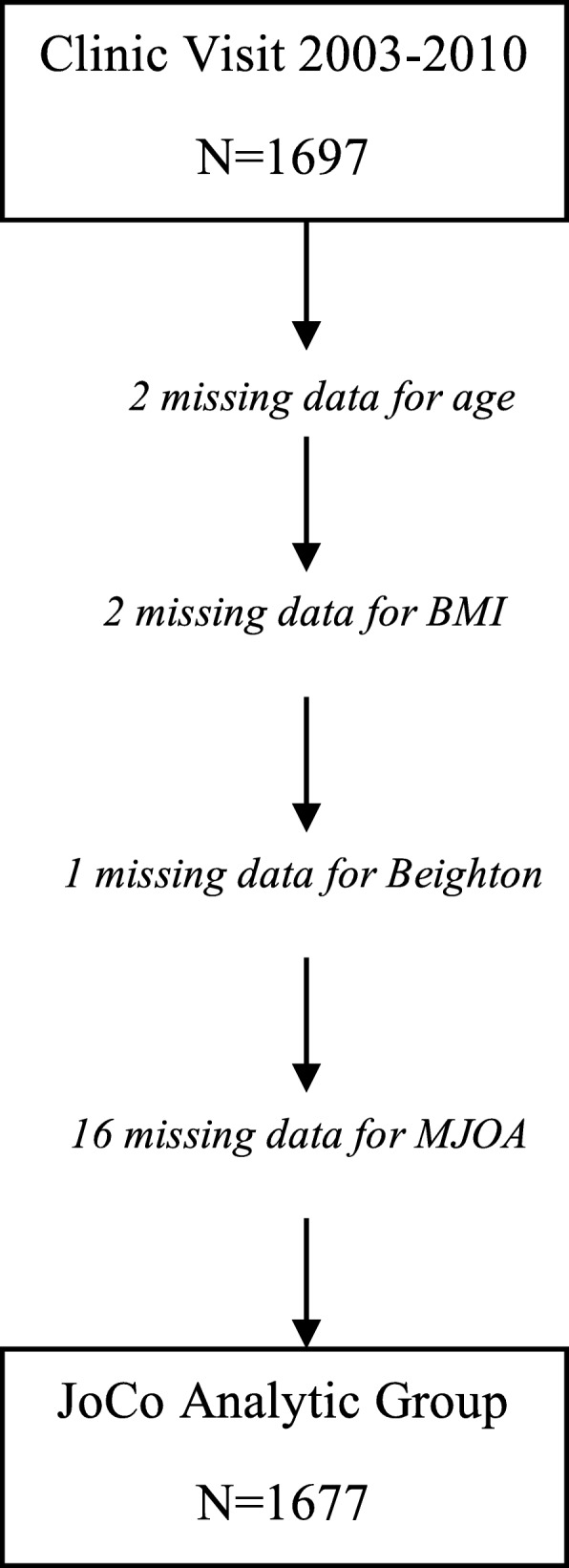
JoCo OA participants with data for analyses

**Fig. 2:**
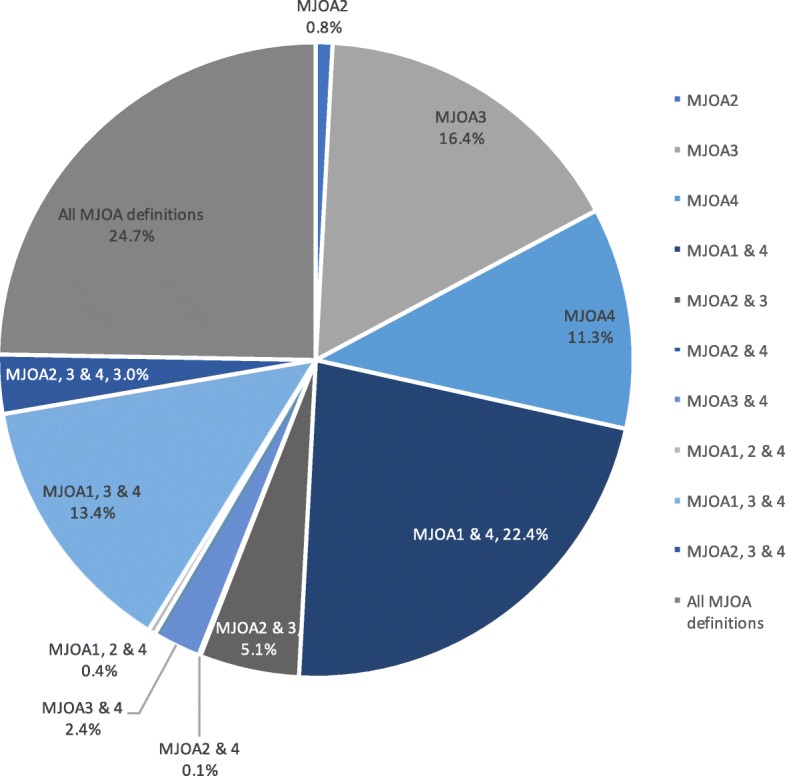
Percentage of those meeting the 4 definitions of radiographic MJOA and their combinations among JoCo OA participants with MJOA (*n* = 1064). Another 613 participants (36.5% of the sample) had no radiographic MJOA, and several combinations were not seen in this cohort (i.e., MJOA-2 only, MJOA-1&2 or 2&3, and MJOA-1,2&3)

There were 1697 participants with Beighton and any OA data available for analyses. Participants with missing values for age (*n* = 2), BMI (n = 2), Beighton (*n* = 1) or MJOA (*n* = 16) were excluded (total *n* = 20), leaving 1677 participants for these analyses (Fig. 1). The mean age of the overall cohort was 68.6 (SD 9.1) years, mean BMI was 31.5 (SD 7.2) kg/m^2^, with 68% women and 31% African Americans. Joint hypermobility was relatively infrequent, with 3.9% of participants (*n* = 65) with a Beighton score ≥ 4 and 8.9% (*n* = 150) with a score ≥ 3. Frequencies of the 4 MJOA definitions within the overall JoCo cohort ranged from 13 to 49% (Table 1). Among those meeting at least one definition of radiographic MJOA (*n* = 1064), about a third met criteria for one (*n* = 303; 28.5%) or two (*n* = 319; 30.0%) definitions, 16.8% (*n* = 179) for 3 definitions, and nearly a quarter met criteria for all four definitions (*n* = 263; 24.7%, Fig. 2).

**Table 1:** Frequencies (%) of multiple joint osteoarthritis (MJOA) in the Johnston County OA Project cohort

Definition(*n* = 1677)^a^	Frequency, n (%)	
	rOA	sxOA
MJOA-1: > 1 IP node and > 2 other sites (hip, knee, spine)	648 (39)	433 (26)
MJOA-2: > 2 IP, > 1 CMC, and knee or hip	363 (22)	225 (13)
MJOA-3: > 5 sites (DIP, PIP, CMC, hip, knee, spine)	693 (41)	426 (25)
MJOA-4: > 2 lower body joint sites (hip, knee, spine)	831 (49)	499 (30)

*rOA* Radiographic osteoarthritis, *sxOA* Symptomatic osteoarthritis, *IP* Interphalangeal joint, *CMC* Carpometacarpal joint, *DIP* Distal interphalangeal joint, *PIP* Proximal interphalangeal joint

^a^one participant could meet more than one definition

Descriptive characteristics of the participants included in the hypermobility analyses by radiographic MJOA status are detailed in Table 2. Overall, compared to those without MJOA, those with MJOA did not differ by BMI but were older, more often women than men and more often white than African American. The only exception to this was in MJOA-4 analyses, wherein there was no statistically significant difference in disease frequency based on sex or race.

**Table 2:** Characteristics of the sample by radiographic multiple joint osteoarthritis (MJOA) definition (*N* = 1677)

	MJOA-1		MJOA-2		MJOA-3		MJOA-4	
	No	Yes	No	Yes	No	Yes	No	Yes
Age (Mean, SD) years	66.8 (8.7)	71.6 (8.9)*	67.0 (8.7)	74.3 (8.2)*	65.4 (8.1)	73.2 (8.4)*	66.2 (8.6)	71.1 (8.9)*
Body Mass Index (Mean, SD) kg/m^2^	31.5 (7.6)	31.5 (6.5)	31.5 (7.2)	31.1 (6.9)	32.0 (7.5)	30.7 (6.7)*	31.2 (7.7)	31.8 (6.6)
Women, N (%)	665 (64.6)	468 (72.2)*	852 (65.0)	278 (76.6)*	623 (63.2)	510 (73.7)*	564 (66.4)	569 (68.8)
African American, N (%)	346 (33.6)	177 (27.3)*	450 (34.4)	72 (19.8)*	396 (40.2)	127 (18.4)*	264 (31.1)	259 (31.3)
Beighton score > 4, N (%)	55 (5.3)	10 (1.5)*	49 (3.7)	16 (4.4)	38 (3.9)	27 (3.9)	34 (4.0)	31 (3.7)
Beighton score > 3, N (%)	115 (11.2)	35 (5.4)*	123 (9.4)	26 (7.2)	97 (9.8)	53 (7.7)	90 (10.6)	60 (7.3)*

*statistically significant difference between the MJOA and no MJOA groups (*p* < .05)

The aOR and corresponding 95% CIs comparing the odds of meeting different MJOA definitions by Beighton criteria are shown in Tables 3 (rOA) and 4 (sxOA). With some exceptions, hypermobility as defined by varying Beighton cutoffs was generally associated with lower odds of both radiographic and symptomatic MJOA; not all associations were statistically significant.

**Table 3:** Associations between Beighton Score and radiographic multiple joint osteoarthritis (MJOA)

Beighton Measure	n with characteristic/N (%) analyzed	Adjusted Odds Ratio (95% Confidence Interval)			
		MJOA-1	MJOA-2	MJOA-3	MJOA-4
Beighton > 4	65/1677 (3.9)	0.26 (0.13–0.52)^a^	1.21 (0.65–2.27)	0.92 (0.52–1.62)	1.02 (0.60–1.72)
Beighton > 3	150/1677 (8.9)	0.45 (0.30–0.68)^a^	0.78 (0.48–1.25)	0.70 (0.47–1.05)	0.73 (0.51–1.05)
Knee hypermobility	33/1642 (2.0)	0.77 (0.36–1.65)	0.61 (0.20–1.80)	0.56 (0.24–1.31)	0.96 (0.47–1.94)
Trunk hypermobility	97/1662 (5.8)	0.80 (0.50–1.26)	0.78 (0.43–1.41)	0.82 (0.50–1.33)	0.77 (0.50–1.19)
Elbow hypermobility	74/1670 (4.4)	0.34 (0.19–0.62)^a^	1.09 (0.61–1.94)	1.03 (0.61–1.73)	0.81 (0.50–1.33)
Fifth finger hypermobility	1108/1670 (66.3)	0.80 (0.64–1.00)	0.86 (0.65–1.12)	1.02 (0.80–1.30)	0.93 (0.75–1.16)
Thumb hypermobility	26/1670 (1.6)	0.59 (0.23–1.53)	1.21 (0.42–3.50)	0.60 (0.23–1.59)	1.33 (0.59–3.03)

^a^statistically significant adjusted odds ratio; all models adjusted for age, body mass index, sex, and race

**Table 4:** Associations between Beighton score and symptomatic multiple joint osteoarthritis (MJOA)

Beighton Measure	n with characteristic/N (%) analyzed	Adjusted Odds Ratio (95% Confidence Interval)			
		MJOA-1	MJOA-2	MJOA-3	MJOA-4
Beighton > 4	65/1677 (3.9)	0.42 (0.20–0.87)^a^	0.72 (0.31–1.64)	1.27 (0.71–2.28)	0.92 (0.52–1.63)
Beighton > 3	150/1677 (8.9)	0.58 (0.37–0.91)^a^	0.54 (0.28–1.01)	0.89 (0.58–1.37)	0.81 (0.54–1.22)
Knee hypermobility	33/1642 (2.0)	0.84 (0.36–1.98)	0.23 (0.03–1.75)	1.11 (0.48–2.57)	1.29 (0.61–2.71)
Trunk hypermobility	97/1662 (5.8)	0.72 (0.42–1.23)	0.53 (0.24–1.18)	0.61 (0.34–1.09)	0.61 (0.35–1.04)
Elbow hypermobility	74/1670 (4.4)	0.65 (0.36–1.16)	0.95 (0.48–1.88)	1.53 (0.91–2.58)	1.01 (0.60–1.70)
Fifth finger hypermobility	1108/1670 (66.3)	0.66 (0.52–0.84)^a^	0.72 (0.53–0.99)^a^	0.91 (0.70–1.17)	0.75 (0.60–0.94)^a^
Thumb hypermobility	26/1670 (1.6)	0.83 (0.30–2.30)	0.30 (0.04–2.34)	0.43 (0.12–1.54)	1.42 (0.59–3.41)

^a^statistically significant adjusted odds ratio; all models adjusted for age, gender, race, body mass index, and baseline visit

Participants with joint hypermobility were almost 75% less likely to have MJOA-1 (aOR 0.26, 95%CI 0.13–0.52, Table 3). However, no statistically significant associations were identified for the other MJOA definitions. The sxOA definitions followed a similar trend, such that those with joint hypermobility were nearly 60% less likely to have MJOA-1 (aOR 0.42, 95%CI 0.20–0.87, Table 4).

We also assessed for relationships between hypermobility at individual sites (knee, trunk, elbow, fifth finger, thumb) and each MJOA definition (shown in the lower rows of Tables 3 and 4). Four of these subgroups—trunk, elbow, knee, and thumb—had small sample sizes (*n* < 100). Many more participants had fifth finger hypermobility (*n* = 1108) than had hypermobility at any other individual joint site. Overall, the pattern of association of MJOA and individual site hypermobility was consistent with findings for generalized hypermobility. Namely, although most of the associations did not attain statistical significance, hypermobility at individual joints was associated with lower odds of radiographic MJOA across definitions. An exception to this trend was that for rOA, elbow hypermobility was statistically significantly associated (aOR 0.34, 95%CI 0.19–0.62) with decreased odds of MJOA-1 (> 1 IP node and > 2 other sites out of hip, knee, and spine).

For sxOA and site-specific hypermobility, trunk hypermobility was associated with decreased odds of symptomatic MJOA-4 before adjustment (OR 0.56, 95%CI 0.33–0.94), but the association attenuated slightly after adjustment (aOR 0.61, 95% CI 0.35–1.04). Hypermobility of the fifth finger was associated with 25–34% lower odds of symptomatic MJOA-1, − 2, − 4 (Table 4).

## Discussion

In this community-based cohort of middle-aged to older adults, hypermobility was generally associated with decreased odds of MJOA across most definitions, although not all relationships were statistically significant. Notably, this relationship was found for both radiographic and symptomatic MJOA definitions. For MJOA-1, generalized joint hypermobility was statistically significantly associated with lower odds of radiographic and symptomatic MJOA. Hypermobility at individual joint sites followed a similar pattern but with inconclusive results likely due in part to small cell sizes because of the low prevalence of joint hypermobility. Joint hypermobility was less common in our cohort (< 10%) than reported in other studies, which may be due to the high frequency of OA among our participants, particularly in the joints that are tested by the Beighton criteria. A joint that might have once been hypermobile could present as not-hypermobile on the Beighton criteria when OA is present.

Our group is the first, to our knowledge, to assess literature-based definitions of MJOA for associations with hypermobility, so we cannot directly compare findings for MJOA across studies. Other published cross-sectional studies have suggested an inverse relationship between joint hypermobility and OA. In the large family-based CARRIAGE study (African American and Native American heritage, *n* = 280), hand and knee hypermobility were significantly associated with a lower prevalence of clinical OA in these joints [17]. Kraus et al. found decreased odds of PIP rOA among those with generalized hypermobility in the GOGO cohort (*n* = 1043, [41]). In our recent work examining the JoCo OA, GO, and GOGO cohorts, participants who completed the trunk flexion maneuver, suggestive of a flexible spine and hamstrings, were less likely to have rOA of the lumbar spine or facet joints; the directionality of this association remains unclear [19].

In contrast, a number of studies reported an increased risk of OA in association with hypermobility. One clinic-based study of female patients (*n* = 100) reported a statistically significant association between sxOA in at least 3 sites and generalized or site-specific hypermobility as defined by Beighton [14]. A 2016 clinical study with 503 Turkish participants found both generalized hypermobility and knee hypermobility to be associated with knee rOA [42]. Lastly, a clinical study in an Icelandic cohort by Jonsson et al. produced mixed findings; generalized hypermobility was associated with 1st CMC clinical OA, yet was inversely associated with hand IP clinical OA (*n* = 200) [15].

These conflicting findings could be due to a few factors. First, multiple outcomes were assessed across studies, including both radiographic and symptomatic OA, and joint symptoms, reducing comparability of results. Second, samples ranged widely in size and source, further reducing generalizability across studies. From a genetic standpoint, joint hypermobility and OA appear to be linked, potentially by abnormalities in collagen contributing to both conditions, as observed in reports showing a higher likelihood of OA among individuals with collagen diseases, like Ehlers- Danlos syndrome [43]. From a biomechanical standpoint, hypotheses exist in the literature for both possibilities of a positive and negative correlation of hypermobility with MJOA. Hypermobility has been hypothesized to be positively associated with MJOA through the mechanism of joint malalignment and injury due to abnormal forces at the joint [7, 8]. Two studies showed that greater loading of foot structures (i.e., greater midfoot peak pressure and maximum force values at the midfoot and hallux) was associated with more severe joint hypermobility [44, 45]. Conversely, it has been proposed that hypermobility may cause pain- or joint instability-induced moderation of activity that may in fact reduce OA risk [17].

A strength of our study design was our use of a large, community-based sample including African American and white men and women. Our consideration of multiple joint sites in data analysis was unique and was supported by a formal systematic review conducted prior to data analysis to derive multiple evidence-based MJOA definitions [36]. Finally, consideration of both rOA and sxOA allowed a greater breadth of understanding of the condition and added to the clinical relevance of the findings.

Limitations of this study include its cross-sectional nature, such that our analyses were restricted to MJOA frequency, without consideration of incidence, progression, or causal associations. The included JoCo participants were all above 45 years old with a mean age of 69 years. Individuals in this cohort who had joint hypermobility when they were younger may be less likely to present with hypermobile joints at the time of the research clinic visit because of aging-related changes to the joint or the development of OA, and thus, their joint hypermobility status may be misclassified. Further, compared to participants without MJOA, those with MJOA were generally older, and for MJOA-1 through − 3 were more likely to be women and white. Thus, we cannot generalize our results to other age groups men, or other racial/ethnic groups. The Beighton score for hypermobility is itself a limitation. As the Beighton score only assesses 5 joint sites, this limits comparison between hypermobility and MJOA measures. For instance, some of our MJOA definitions included a requirement for IP involvement, which, aside from the tests for 1st and 5th digit hypermobility, is not well-represented in the Beighton criteria. Also, hip hypermobility is assessed as part of the trunk maneuver—along with spine mobility and hamstring flexibility—thus preventing independent assessment of this joint. This, along with the small sample sizes for those with hypermobility in individual joints, limited our ability to draw meaningful conclusions from the relevant data at each joint site. The associations overall were modest and should be considered in the context of the overall sample size and number of comparisons presented.

Validation of the MJOA definitions used in these analyses in other cohorts is needed to determine generalizability. Additionally, a better appreciation of hypermobility at individual joint sites, including hip and specific IP measures, could improve understanding of site-specific relationships between MJOA and hypermobility. Lastly, more research into the biomechanical effects of hypermobility on joint physiology would aid our understanding of a potential mechanistic link between hypermobility and MJOA and guide further study of these conditions.

## Conclusion

In summary, in this large community-based cohort of adults, joint hypermobility was inversely associated with at least one definition of MJOA and was not positively associated with either radiographic or symptomatic MJOA by any definition. To assess their validity, it would be of value for MJOA definitions used here to be applied to other large cohorts. Longitudinal studies are needed to determine the contribution of hypermobility to the incidence and progression of MJOA outcomes over the life course in cohorts that include younger adults.

## Abbreviations

aOR: Adjusted odds ratio
BMI: Body mass index
CI: Confidence interval
CMC: Carpometacarpal
DIP: Distal interphalangeal
IP: Interphalangeal
JoCo OA: Johnston County Osteoarthritis
KL: Kellgren-Lawrence
MJOA: Multiple joint osteoarthritis
OA: Osteoarthritis
OR: Odds ratio
PIP: Proximal interphalangeal
rOA: Radiographic osteoarthritis
SD: Standard deviation
sxOA: Symptomatic osteoarthritis
